# Mathematical Formulation of Energy Minimization – Based Inverse Optimization

**DOI:** 10.3389/fonc.2014.00181

**Published:** 2014-07-18

**Authors:** Ivaylo B. Mihaylov

**Affiliations:** ^1^Department of Radiation Oncology, University of Miami, Miami, FL, USA

**Keywords:** dose, volume, mass, energy, integral dose, inverse optimization

## Abstract

**Purpose:** To introduce the concept of energy minimization-based inverse optimization for external beam radiotherapy.

**Materials and Methods:** Mathematical formulation of energy minimization-based inverse optimization is presented. This mathematical representation is compared to the most commonly used dose–volume based formulation used in inverse optimization. A simple example on digitally created phantom is demonstrated. The phantom consists of three sections: a target surrounded by high and low density regions. The target is irradiated with two beams passing through those regions. Inverse optimization with dose–volume and energy minimization-based objective functions is performed. The dosimetric properties of the two optimization results are evaluated.

**Results:** Dose–volume histograms for all the volumes of interest used for dose optimization are compared. Energy-based optimization results in higher maximum dose to the volumes that are used as dose-limiting structures. However, the average and the integral doses delivered for the volumes outside of the target are larger with dose–volume optimization.

**Conclusion:** Mathematical formulation of energy minimization-based inverse optimization is derived. The optimization applied on the digital phantom shows that energy minimization-based approach tends to deliver somewhat higher maximum doses compared to standard of care, realized with dose–volume based optimization. At the same time, however, the energy minimization-based optimization reduces much more significantly the average and the integral doses.

## Introduction

The basic principle of external beam radiotherapy involves irradiation from a number of different directions (cross-firing) with beams of uniform or non-uniform energy fluences (intensities). The aim of this arrangement is to deliver a high dose to the target volume, while delivering as low doses as possible to the surrounding normal tissues. Radiotherapy dose calculations are based upon the following framework. CT derived attenuation coefficients (or Hounsfield Units) are mapped to electron density through a calibration procedure. The electron density (which scales with physics density of the material) governs the number of photon Compton interactions. The electrons, set in motion due to those Compton interactions, lead to ionizations, which affect the underlying biological response in the living cells and in particular lead to cell kill.

By its very definition dose is the radiation energy imparted per unit mass of material (Gy = J/kg). The volume integral of the deposited dose therefore has units of energy and is also known as “integral dose.” Alternatively in the discrete case applicable in radiotherapy, if dose is multiplied by mass on a dose voxel-by-voxel basis, and a summation over all dose voxels within a volume of interest (VOI) is performed, then the total energy imparted to that VOI would be obtained. It is often mentioned in the literature that the large number of beamlets and monitor units used in intensity modulated radiotherapy (IMRT) leads to an increase in integral dose compared to conformal radiotherapy (3DCRT) (1, 2). Furthermore, it is also commonly assumed that higher-energy photon beams substantially reduce the integral dose to normal tissue (3). However, an alternative hypothesis suggests that the total energy deposited in a patient during irradiation is relatively independent of treatment planning parameters, with some reduction of integral dose with higher-energy beams (4–6).

The use of integral dose in plan evaluation has been underutilized, although it could be a valuable metric, especially in cancer cases with curative intent for patients with long life expectancy. Therefore, it is somewhat surprising that so far integral dose (or total energy) minimization has been neglected in radiotherapy plan optimization. The aim of this work is to shed some light on the mathematical basis for the utilization of integral dose minimization in IMRT, and to present its application on a simple example.

## Mathematical Framework of Integral Dose Minimization-Based Inverse IMRT Optimization

Energy minimization approaches are commonly encountered in physics where the solutions of many problems are very often based on this fundamental principle. The total energy imparted on an anatomical organ of interest is given by the integral dose and can be expressed according to Eq. 1,

(1) $$\begin{array}{llll} E_{\text{total}} & =I=\sum_{i=1}^{N}d_{i}m_{i}=\sum_{i=1}^{N}d_{i}\rho_{i}v_{i}\; & & \;\\ & =\sum_{i=1}^{N}\frac{E_{i}}{\rho_{i}v_{i}}\rho_{i}v_{i}=\sum_{i=1}^{N}E_{i} \end{array}$$

where *d_i_, m_i_, ρ_i_*, and *v_i_* are the dose, mass, density, and volume of dose voxel *i*, respectively. The summation is over all dose voxels contained in the volume of the organ, and *E_i_* is the energy imparted on voxel *i*. Mathematical incorporation of Eq. 1 in inverse optimization can be achieved after rewriting the representation as described in Eq. 2,

(2) $$F^{j}=\frac{1}{E_{\text{desired}}}\sum_{i\in V}d_{i}m_{i}$$

where *F^j^* is the *j^th^* objective function, *E*_desired_ is the desired integral dose, and the summation *i* is over all voxels within the volume of a given anatomical structure. Note that for each anatomical structure, where integral dose minimization is required, only one objective can be specified, as opposed to dose–volume histogram (Dvh), or generalized equivalent uniform dose optimization (gEUD-based), where multiple objectives can be specified for the same anatomical structure (7–10). The minimization of course is applicable to organs at risk (OARs), where it is required that the delivered dose is as low as possible. The normalization to the desired integral dose *E*_desired_ is performed such that a composite objective function (cf. Eq. 3) can be constructed, where individual objective functions *F^j^* can be expressed in terms of other dose representations (7, 11, 12).

(3) $$F=\sum_{j=1}^{M}F^{j}$$

Each individual term in the summation in Eq. 2 is always positive by construction since dose and mass can only be positive variables. Therefore, there is no need to introduce a quadratic form which requires minimization, as it is the case in Dvh- or gEUD-based optimizations (7, 12–14).

## Example

**Figure 1:**
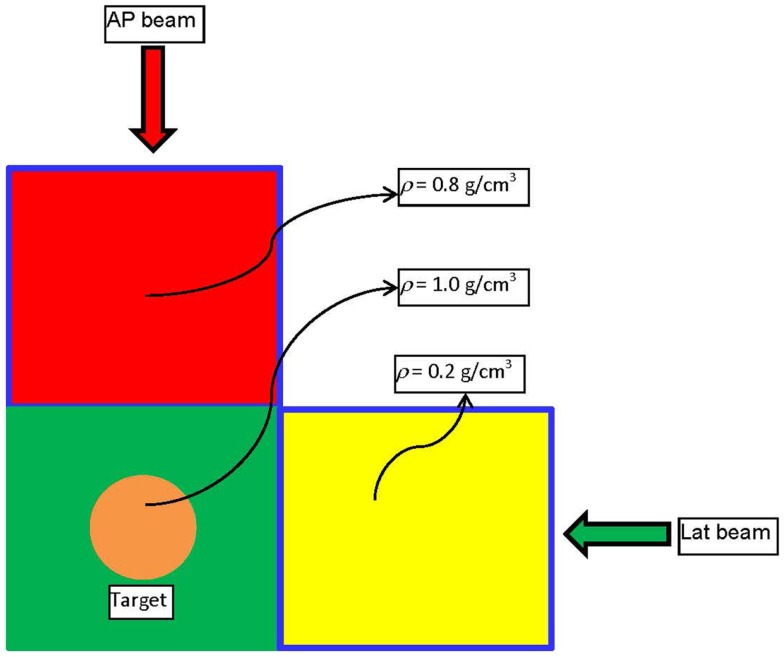
**Schematic diagram of the phantom and the beam arrangement used to demonstrate the basic physics principle of Energy-based inverse optimization**.

**Figure 2:**
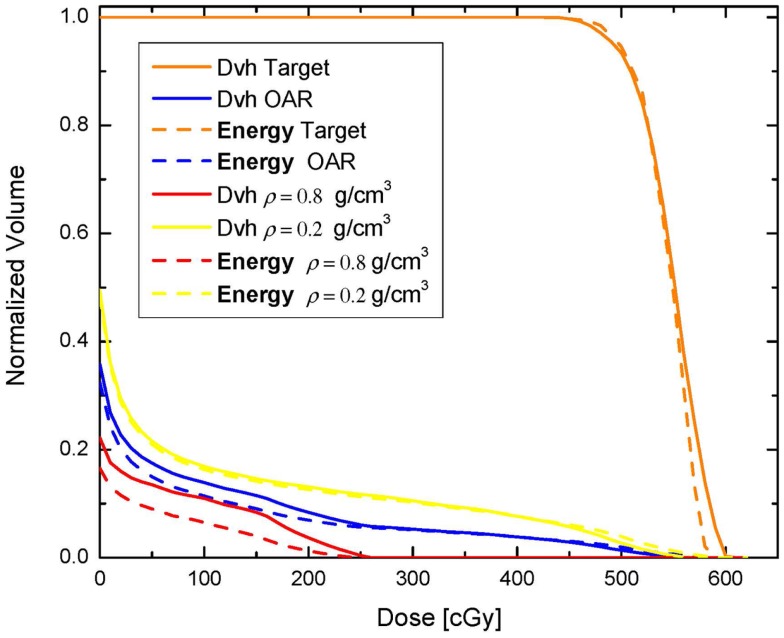
**Comparison between dose-volume histograms resulting from Dvh-based and Energy-based optimization**.

An example is presented to illustrate the basic points of the derived framework for energy minimization-based optimization, and to outline the differences with Dvh-based optimization. Figure 1 depicts a digital phantom in an axial view, on which the example will be illustrated. The phantom consists of three 10 cm × 10 cm × 10 cm cubical VOIs with densities of 0.2 (yellow), 0.8 (red), and 1.0 (green) g/cm^3^. In the middle of the green VOI, there is a cylindrical target 3 cm in diameter and 3 cm in length. The high (red) and low (yellow) density regions are combined to form an “OAR” to which the dose is to be minimized through an inverse optimization. The target is irradiated with an anterior–posterior (AP) and a lateral (Lat) beam centered on the geometric center (isocenter) of the target. The two beams – AP and Lat are allowed to have only one IMRT segment each. Two IMRT plans are generated with that beam configuration. In the first plan, the cost function for OAR dose optimization is constructed according to Eq. 4,

(4) $$F^{j}=\sum_{i\in V}{\left(\frac{d_{i}-d^{j}}{d^{j}}\right)}^{2}\Delta v_{i}$$

where *V* denotes the volume of the OAR for which *F ^j^* is evaluated, *d_i_* is the dose in voxel (3D volume element) *i, d ^j^* is the desired dose in each voxel, and *v_i_* is the normalized (with respect to the entire OAR volume) voxel volume (12). In the second plan, the OAR dose optimization is based on Eq. 2. Those two optimization schemes are termed Dvh-based and Energy-based, respectively. The inverse optimization was performed with a gradient decent method (7, 12, 15–18) and was realized similarly to what other investigators have proposed (7). With each optimization the dose to the OAR is iteratively decreased until the standard deviation of the dose across the target reaches 6% of the prescription dose, i.e., no more than 30 cGy. The Dvhs of the two optimization approaches are presented on Figure 2. In addition, maximum and integral doses for the OAR, as well as the high and the low density VOIs are presented in Table 1. As can be noted on the figure and the table the high dose tails to the low density region (yellow) and the OAR (blue) are higher with Energy-based optimization, while the entire Dvh for the higher density region is lower than in the case of Dvh-based optimization. Therefore, in solving the global optimization problem, it seems that Energy-based optimization delivers more dose through the lower density region. Calculated doses to 1% of the OAR (as surrogate for maximum dose) are 541 and 520 cGy with Energy- and Dvh-based optimizations, respectively. This difference indicates ~4% difference in the maximum dose to the OAR. However, the average doses delivered to the OAR are 45 and 50.2 cGy, respectively, indicating that the Energy-based based optimization delivers 11.5% lower average dose to the OAR.

**Table 1:** **Integral doses and doses to 1% volumes of OAR, high density, and low density VOIs**.

	OAR		High density VOI		Low density VOI	
	Dvh-based	Energy-based	Dvh-based	Energy-based	Dvh-based	Energy-based
*D*_max_ (cGy) (1% volume)	520	541	248	216	540	564
Integral dose (J)	0.342392	0.26327	0.197955	0.120046	0.144438	0.143224

*The doses to 1% volumes of the different regions are used as surrogates for maximum doses*.

Furthermore, comparison of the integral doses to the irradiated volume, excluding the target, is evaluated. All three VOIs red, green, and yellow from Figure 1 are combined in a single structure and the integral dose to that volume is evaluated. The total imparted energy to that structure with Dvh-based optimization is 1.09977 J, while with Energy-based optimization the imparted energy is 0.941815 J. Therefore, in this very simple scenario the Energy-based inverse optimization results in an integral dose reduction to the entire volume in excess of 16%. If only the integral doses to the OAR are considered (combination of red and yellow VOIs) then the total energy imparted to this region with Energy-based optimization is ~30% lower, as can be concluded from Table 1.

## Conclusion

A new approach based on energy-reduction inverse optimization was outlined. The mathematical framework for this optimization was defined. The energy-reduction approach was compared to the standard of care in inverse IMRT optimization, realized through Dvh-based optimization. Both optimization techniques were applied to a simple digital phantom for a very simple beam geometry, and their dosimetric properties were compared. Energy-reduction approach resulted in lower average and integral doses to the volumes surrounding the irradiated target.

## Conflict of Interest Statement

The author declares that the research was conducted in the absence of any commercial or financial relationships that could be construed as a potential conflict of interest.
